# Icu and long-term mortality analisis of hematology patients admitted in icu. a 7 years review from a single spanish center

**DOI:** 10.1186/2197-425X-3-S1-A244

**Published:** 2015-10-01

**Authors:** R Garcia Gigorro, A Jiménez-Ubieto, H Marín, G Morales-Varas, C Grande-García, JC Montejo-González

**Affiliations:** Intensive Care, Hospital 12 de Octubre, Madrid, Spain; Hematology, Hospital 12 de Octubre, Madrid, Spain

## Intr

Improved survival in patients with haematological malignancy (HM) admitted to the intensive care unit (ICU) has largely been reported in uncontrolled cohorts. Newly diagnosed patients should be admitted, since their prognosis is still to be defined. Nevertheless the admission of the remaining patients remains a matter of substantial controversy.

## Objectives

To analyse the survival of HM patients admitted to ICU over a 7-year period (from 2008 to 2014).

## Methods

We conducted a detailed retrospective study of sequential adult ICU admissions with HM in a single centre, considering numerous variables with regard to their influence on ICU and mortality. Overall survival (OS) was defined as the time from ICU admission to death from any cause, and surviving patients were censored at last follow-up. OS were calculated using the Kaplan-Meier method estimates and the differences assessed by the log-rank test. All *P-*value less than 0.05 were considered significant.

## Results

Overall, 67 HM patients were included, 62% were male, with a median age of 59 years (IQR: 19-82). The median APACHE II was 22 (IQR: 12-49). The hematologic diagnosis was as follow: 25 lymphomas (37%), 9 Multiple Myelomas (13%), 6 chronic lymphocytic leukaemia (9%) and 28 acute leukaemia or myeloproliferative or myelodisplasic syndromes in acute phase (41%). Disease status at the moment of UCI admission was: 19 (28%) complete response (CR), 24 (36%) newly diagnosis disease and 24 (36%) active disease (of those, 18 patients were refractory to disease specific treatment and 6 patients were chemosensitive); 67% of patients were on active oncologic treatment. Principal ICU diagnosis was sepsis (68%). Median number of organ failure were 2 (IQR: 0-4), and up to 36% of patients presented neutropenia at the moment of UCI admission. Median ICU length of stay was 7 days (IQR: 1-48) and hospital of stay 38 days (IQR: 8-109). ICU mortality was 46% (25% of patients died the first day of ICU admission, and 75% died within the first week).

**Figure 1:**
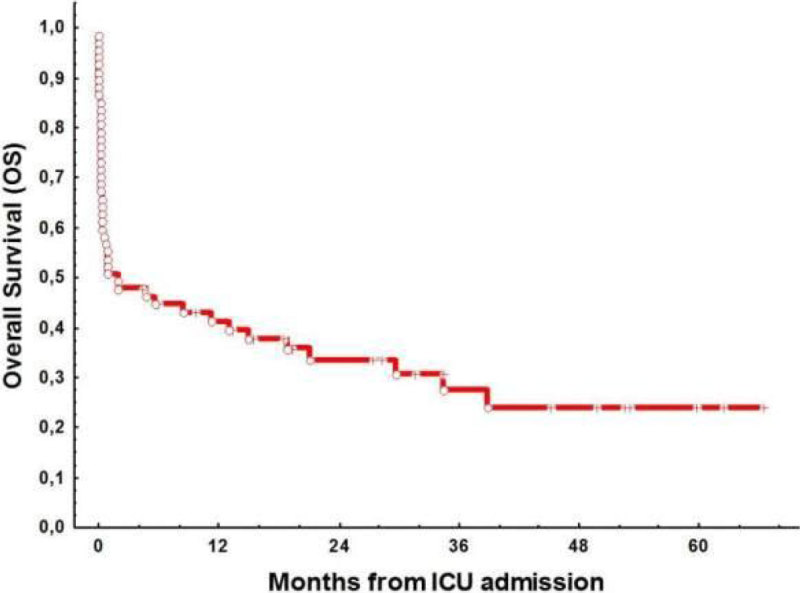
**Overall Survival.**

**Figure 2:**
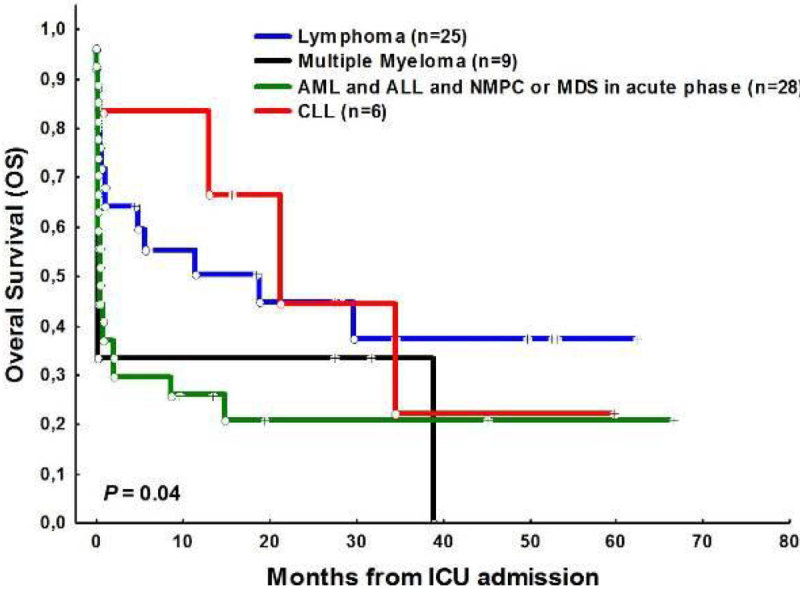
**OS and type of HM in ICU admission.**

**Figure 3:**
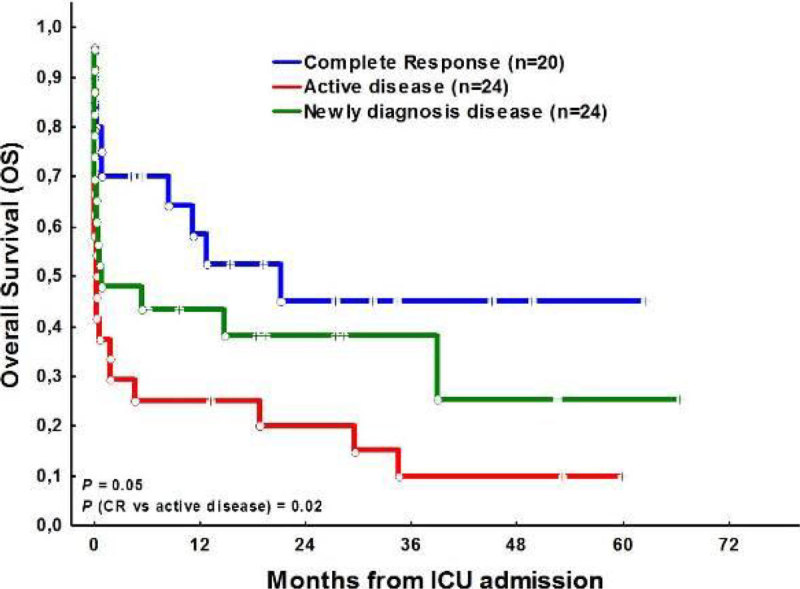
**OS and type of response in ICU admission.**

The rates for ICU, 1-month, 6-month and 12 month mortality were 48%, 55% and 66%, respectively. With a median follow up of 18.5 months (IQR: 8-66), OS was 31%. Figure 1; Hematologic diagnosis (*P* = 0.04) Figure 2; status of the disease at the moment of ICU admission (*P* = 0.01) Figure 3; and the number of organ failure (≤ 2: 67% vs ≥ 3: 28%, *P* = 0.009) were the most powerful predictor variables associated with an increased mortality.

## Conclusions

OS of our HM patients is not worse than that recently reported from specialist units.The decision for or against ICU admission of patients with HM should become dependent of the underlying malignant disease and especially with the status of the disease at the moment of admission. Almost 50% of survivors are still alive one year after ICU admission, suggesting that an important subgroup of HM patients benefit from ICU admission.

